# Dnmt1s in donor cells is a barrier to SCNT-mediated DNA methylation reprogramming in pigs

**DOI:** 10.18632/oncotarget.16507

**Published:** 2017-03-23

**Authors:** Xuexiong Song, Zhonghua Liu, Hongbin He, Jianyu Wang, Huatao Li, Jingyu Li, Fangzheng Li, Zhongling Jiang, Yanjun Huan

**Affiliations:** ^1^ College of Animal Science and Technology, Qingdao Agricultural University, Qingdao, Shandong Province, China; ^2^ College of Life Science, Northeast Agricultural University, Harbin, Heilongjiang Province, China; ^3^ College of Life Science, Shandong Normal University, Jinan, Shandong Province, China; ^4^ Institute of Life Science, Chongqing Medical University, Chongqing, China

**Keywords:** Dnmt1s, DNA methylation reprogramming, somatic cell nuclear transfer, embryo, pig

## Abstract

Low development of somatic cell nuclear transfer embryos could be due to the incomplete DNA methylation reprogramming, and Dnmt1s existing in donor cells may be one cause of this disrupted DNA methylation reprogramming. However, the reprogramming pattern of Dnmt1s and its effect on DNA methylation reprogramming in cloned embryos remain poorly understood. Here, we displayed that along with the significantly higher Dnmt1 expression at the zygotic gene activation stage of cloned embryos, genomic methylation level was markedly upregulated, and the arrested rate was significantly higher compared with their *in vitro* fertilization counterparts. Then, we demonstrated that Dnmt1s, not Dnmt1o, methylation and expression levels in cloned embryos were significantly higher from the 1-cell to 4-cell stage but markedly lower at the blastocyst stage. When Dnmt1s in donor cells was appropriately removed, more cloned embryos passed through the zygotic gene activation stage and the blastocyst rate significantly increased. Furthermore, Dnmt1s knockdown significantly improved itself and genomic methylation reconstruction in cloned embryos. Finally, we found that Dnmt1s removal significantly promoted the demethylation and expression of pluripotent genes in cloned embryos. Taken together, these data suggest that Dnmt1s in donor cells is a critical barrier to somatic cell nuclear transfer mediated DNA methylation reprogramming, impairing the development of cloned embryos.

## INTRODUCTION

Three main approaches including cell fusion, somatic cell nuclear transfer (SCNT) and transcription-factor transduction have been developed to reprogram terminally differentiated somatic cells toward pluripotency, among which, SCNT can induce totipotency to produce cloned animals, with the great application prospect in the fields of agriculture, medicine, species conservation, etc [1–3]. However, to date, the cloning efficiency remains low, limiting SCNT-associated research and application [4–6].

The reason for the low cloning efficiency is considered to be the incomplete epigenetic reprogramming [5], among which, DNA methylation, the most studied epigenetic modification, can reflect the epigenetic reprogramming degree of SCNT embryos [7–9]. It has been shown that DNA methylation reprogramming in most SCNT embryos is aberrant, leading to the continuous expression of tissue specific genes and inefficient activation of genes essential for the embryonic development, thereby reducing the developmental competence of SCNT embryos [5, 8].

Generally, it is thought that the incomplete DNA methylation reprogramming in SCNT embryos can be due to that tissue specific genes in donor cells sustain their fate against nuclear reprogramming induced by oocyte factors [6]. Presently, certain repressive factors, such as Dnmt1, Dnmt3L and H3K9me3, have been identified, but the regulatory mechanism is not well clarified in SCNT embryos [10–12]. As the most studied repressive factor, the maintenance DNA methyltransferase Dnmt1 is thought to be closely associated with the aberrant development of SCNT embryos, however, whether there is a positive relationship between the disrupted expression of Dnmt1 and the incomplete DNA methylation reprogramming still needs to be determined [12, 13].

Furthermore, Dnmt1 gene locus encodes three isoforms by alternative usage of multiple first exons: Dnmt1s (somatic form), Dnmt1o (oocyte specific form) and Dnmt1p (the form in pachytene spermatocytes) [14]. Among these isoforms, Dnmt1o is the only form before zygotic genome activation (ZGA) and Dnmt1s is zygotic origin. As Dnmt1s is expressed in somatic cells, SCNT embryos have to contain the unwanted Dnmt1s before ZGA. Given that the developmental block of SCNT embryos appears at the ZGA stage, it is speculated that Dnmt1s, deriving from donor cells, could probably be the cause [8]. Although previous studies have identified a number of dysregulated genes during ZGA, whether Dnmt1s in somatic cells brings an effect on Dnmt1o and zygotic Dnmt1s, what are the reprogramming patterns of Dnmt1s and Dnmt1o, and how Dnmt1s regulates the development of SCNT embryos remain unknown.

Our previous studies have reported that reducing Dnmt1 expression by epigenetic modification agents or siRNA technology in SCNT embryos improves DNA methylation reprogramming and enhances the embryonic development, whereas Dnmt1o knockdown in MII stage oocytes has no significant effect on the SCNT efficiency, indicating that the disrupted DNA methylation reprogramming in SCNT embryos is closely associated with Dnmt1s in donor cells [15, 16]. To better understand the role of donor cell Dnmt1s in SCNT-mediated DNA methylation reprogramming, we investigated the reprogramming pattern of Dnmt1s and its effect on DNA methylation reprogramming in SCNT embryos. Our results demonstrated that in response to the high Dnmt1 expression, the developmental block and incomplete genomic DNA methylation reprogramming in SCNT embryos occurred during ZGA. Further, the reprogramming pattern of Dnmt1s not Dnmt1o was disrupted in SCNT embryos. Moreover, Dnmt1s knockdown in donor cells improved SCNT-mediated genomic and gene-specific DNA methylation reprogramming and the expression of embryonic development related genes, thereby enhancing the development of SCNT embryos. Overall, Dnmt1s in donor cells is considered to impair the development of SCNT embryos through disrupting DNA methylation reprogramming. This work provides a clear insight into the regulatory mechanism of Dnmt1s in SCNT-mediated DNA methylation reprogramming, and would have important implications in improving cloning efficiency.

## RESULTS

### Abnormal ZGA was associated with the disrupted Dnmt1 expression in 4-cell SCNT embryos

**Figure 1:**
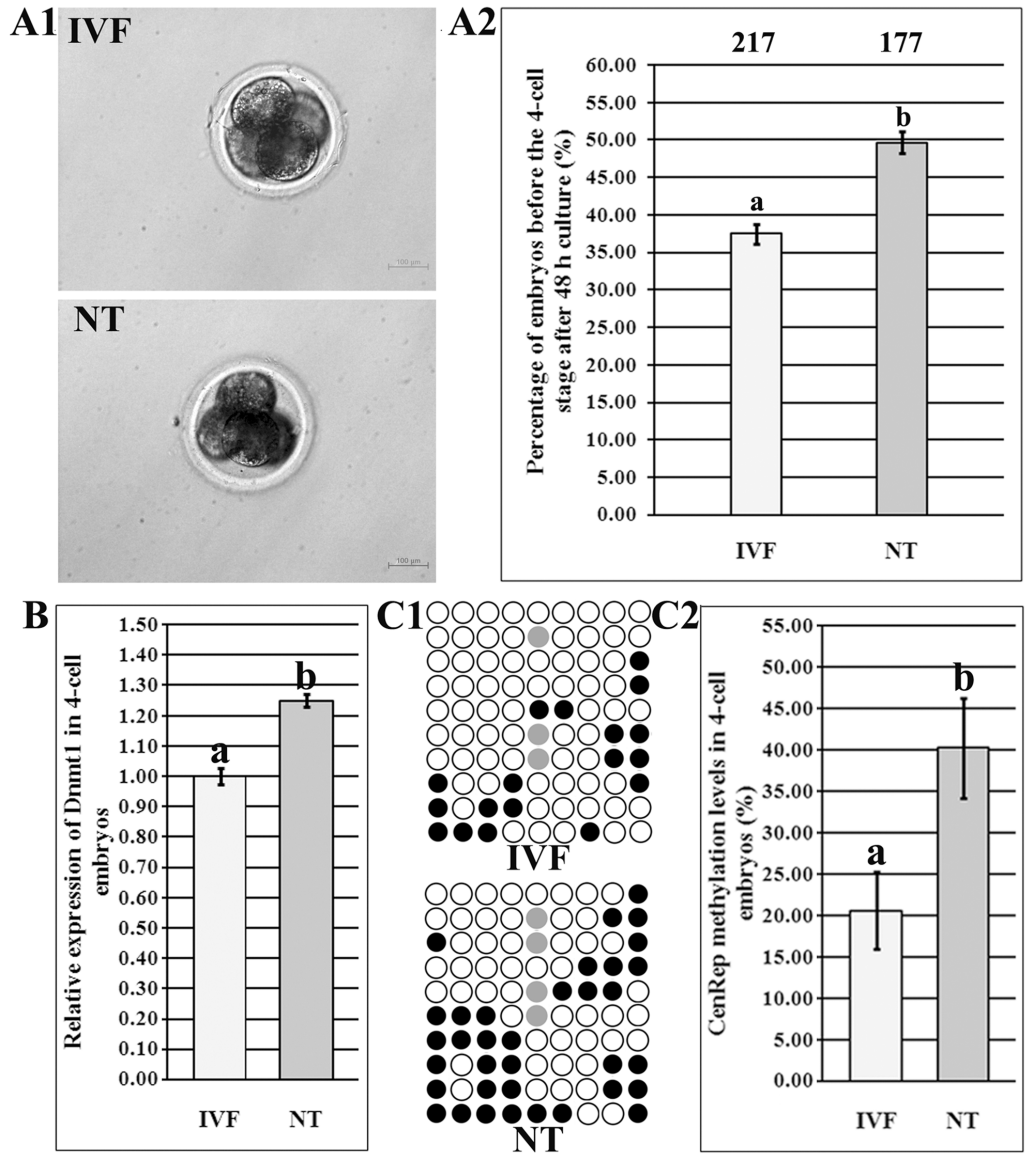
Abnormal ZGA in 4-cell SCNT embryos **(A1)**, the morphology of IVF and SCNT 4-cell embryos, **(A2)**, the percentages of IVF and SCNT embryos arrested before the 4-cell stage after 48 h culture, **(B)**, relative Dnmt1 transcripts in IVF and SCNT 4-cell embryos, **(C1)**, CenRep methylation statuses in IVF and SCNT 4-cell embryos, and **(C2)**, CenRep methylation levels in IVF and SCNT 4-cell embryos. SCNT embryos displayed the developmental block, disrupted Dnmt1 expression and incomplete genomic DNA methylation reprogramming during ZGA. The number of embryos detected was on the top of the column chart. Black or white circles indicate methylated or unmethylated CpG sites, respectively, and gray circles represent mutated and/or SNP variation at certain CpG sites. ^a-b^Values in the same group with different superscripts differ significantly (P<0.05).

Our previous studies have shown that compared with those in IVF embryos, low embryonic development and incomplete DNA methylation reprogramming occurred in SCNT embryos [17, 18]. Here, we further demonstrated that the percentage of SCNT embryos arrested before ZGA was significantly higher than that of IVF embryos during ZGA (Figure 1A1 and 1A2, 49.68% vs 37.50%, P<0.05), and, Dnmt1 expression level was also significantly upregulated in SCNT embryos (Figure 1B, P<0.05). Moreover, SCNT embryos displayed the significantly higher genomic methylation level than IVF embryos during ZGA (Figure 1C1 and 1C2, 40.28% vs 20.56%, P<0.05). Taken together, these results suggested that aberrant Dnmt1 expression may contribute to the incomplete genomic methylation reprogramming, further leading to the abnormal ZGA in SCNT embryos.

### Dnmt1s, not Dnmt1o, displayed the disrupted DNA methylation and expression patterns in SCNT embryos

To determine whether Dnmt1o or Dnmt1s is associated with the abnormal ZGA and low development of SCNT embryos, DNA methylation and mRNA expression patterns of Dnmt1o and Dnmt1s were investigated. The distribution of CpG sites in the upstream of Dnmt1o or Dnmt1s TSS were first analyzed using the MethPrimer program, and 1 CpG island or 2 CpG islands in the Dnmt1o or Dnmt1s upstream was observed, respectively (Supplementary Figure 1A1 and 1B1). Then, the methylation statuses of different regions of Dnmt1o (Region I, Region II, Region III and Region IV) and Dnmt1s (Region I and Region II) in sperms, oocytes (only Dnmt1o expression) and PFFs (only Dnmt1s expression) were examined (Supplementary Figure 1). Compared with that of sperms or PFFs, oocyte methylation level was significantly lower in Region II, Region III or Region IV but not significantly different in Region I of Dnmt1o (Supplementary Figure 1A2 and 1A3, P<0.05), and PFFs methylation level in Region I or Region II of Dnmt1s displayed no significant difference from that of sperms or oocytes (Supplementary Figure 1B2 and 1B3), suggesting that Region II (6 CpG sites), Region III (5 CpG sites) and Region IV (3 CpG sites) of Dnmt1o could represent the differentially methylated region (DMR) of Dnmt1 including Dnmt1s, and the methylation level of Dnmt1 including these three DMRs was negative with Dnmt1o expression but positive with Dnmt1s transcription.

**Figure 2:**
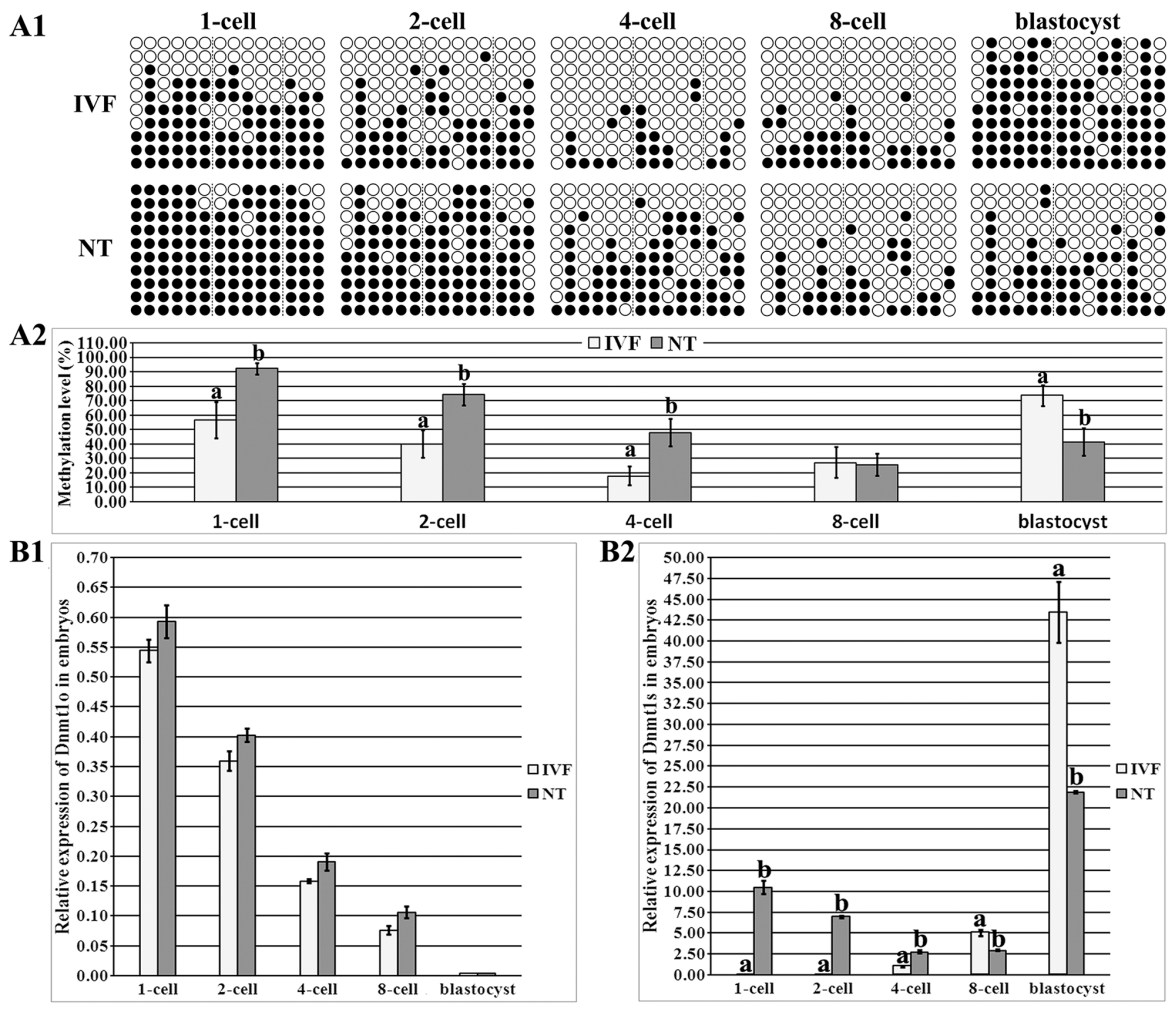
DNA methylation and expression levels of Dnmt1o and Dnmt1s in IVF and SCNT embryos **(A1)**, the methylation statuses of Dnmt1 at 1-cell, 2-cell, 4-cell, 8-cell and blastocyst stages of IVF and SCNT embryos, **(A2)**, the methylation levels of Dnmt1 in the IVF and SCNT groups, **(B1)**, relative Dnmt1o transcripts at the 1-cell, 2-cell, 4-cell, 8-cell and blastocyst stages of IVF and SCNT embryos, and, **(B2)**, relative Dnmt1s expression in the IVF and SCNT groups. SCNT embryos displayed incomplete reprogramming of Dnmt1s methylation and expression. Black or white circles indicate methylated or unmethylated CpG sites, respectively. The transcript abundance in MII oocytes (B1) or IVF 4-cell embryos (B2) was considered to be the control. The data were expressed as mean ± SEM. ^a-b^ Values at a given stage with different superscripts differ significantly (P<0.05).

In IVF embryos, Dnmt1 took on a gradual demethylation from the 1-cell to ZGA stage and remethylation from the ZGA to blastocyst stage, respectively, and Dnmt1 methylation level during ZGA was significantly lower than those in 1-cell and blastocyst embryos (Figure 2A1 and Supplementary Figure 2A, P<0.05). As for the individual Region II, Region III or Region IV in IVF embryos, a similar dynamic to the whole Dnmt1 methylation pattern was observed (Supplementary Figure 2C1). After SCNT (Figure 2A1 and Supplementary Figure 2B), Dnmt1 displayed a gradual demethylation from the 1-cell to 8-cell stage, and an upward trend from the 8-cell to blastocyst stage, and Dnmt1 methylation level during ZGA was significantly lower than that in 1-cell embryos (P<0.05). And, Dnmt1 methylation pattern in the individual Region II, Region III or Region IV was also similar to the overall trend in SCNT embryos (Supplementary Figure 2C2). When comparing the individual developmental stage between SCNT and IVF embryos (Figure 2A1 and 2A2), Dnmt1 took on significantly higher DNA methylation levels from the 1-cell to 8-cell stage and a markedly lower methylation status at the blastocyst stage of SCNT embryos (P<0.05), and, Region II, Region III or Region IV of Dnmt1 also displayed this altered pattern (Supplementary Figure 2C2). Thus, Dnmt1 displayed a pattern of delayed demethylation and failed remethylation in SCNT embryos.

To further identify whether Dnmt1o or Dnmt1s reprogramming pattern is disrupted in SCNT embryos, we examined Dnmt1o and Dnmt1s transcription (Supplementary Figure 3 and Figure 2). In IVF embryos, Dnmt1o transcription significantly and progressively decreased, Dnmt1s expression markedly increased from the 1-cell to blastocyst stage (Supplementary Figure 3B1 and 3B2, P<0.05), and, the ratio of Dnmt1s to Dnmt1o displayed that the main form was Dnmt1o before the 8-cell stage and Dnmt1s in blastocysts, respectively (Supplementary Figure 3B3). In SCNT embryos, Dnmt1o expression and the ratio of Dnmt1s to Dnmt1o took on similar patterns to those in IVF embryos (Supplementary Figure 3B1 and 3B3), while Dnmt1s transcription significantly decreased from the 1-cell and ZGA stage, and increased in blastocysts (Supplementary Figure 3B2, P<0.05). When Dnmt1 expression was compared between IVF and SCNT embryos, no significant differences of Dnmt1o transcription were observed (Figure 2B1), but Dnmt1s expression was significantly upregulated from the 1-cell to ZGA stage and downregulated at the 8-cell and blastocyst stages of SCNT embryos (Figure 2B2, P<0.05). These results demonstrated that Dnmt1s expression was aberrant in SCNT embryos, further supporting that Dnmt1s methylation reprogramming was incomplete in SCNT embryos. Thus, the upregulated Dnmt1s transcription resulting from its high DNA methylation could lead to abnormal ZGA, and further, inefficient expression of zygotic Dnmt1s would probably result in the poor quality of SCNT blastocysts (Supplementary Figure 4). Overall, DNA methylation reprogramming and expression patterns of Dnmt1s were disrupted in SCNT embryos, blocking the ZGA and subsequent embryonic development.

### Dnmt1s knockdown in donor cells reduced the developmental arrest and improved the subsequent development of SCNT embryos

**Figure 3:**
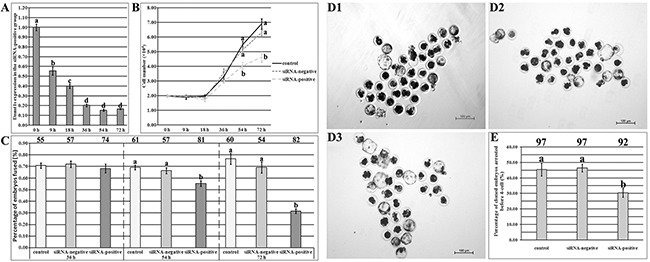
The development of SCNT embryos derived from donor cells with Dnmt1s knockdown **(A)**, Dnmt1s expression in PFFs at 9 h, 18 h, 36 h, 54 h or 72 h of Dnmt1s-siRNA transfection. **(B)**, cell number of PFFs at 9 h, 18 h, 36 h, 54 h or 72 h of Dnmt1s-siRNA transfection. **(C)**, the fusion rate of SCNT embryos derived from donor cells with 36 h, 54 h or 72 h Dnmt1s-siRNA transfection. The number of reconstructed embryos detected was on the top of the column chart. **(D)**, SCNT blastocysts. **(D1)**, **(D2)** and **(D3)**, SCNT blastocysts derived from donor cells untransfected, transfected with the control siRNA or Dnmt1s-siRNA for 36 h, respectively. **(E)**, the percentage of SCNT embryos arrested before ZGA after donor cells transfected with Dnmt1s-siRNA for 36 h. Dnmt1s knockdown in donor cells improved the development of SCNT embryos. The number of cloned embryos detected was on the top of the column chart. The data were expressed as mean ± SEM. ^a-d^Values in a certain group with different superscripts differ significantly (P<0.05).

To remove Dnmt1s from SCNT embryos before ZGA, Dnmt1s in donor cells should be effectively knocked down. Then, we transfected PFFs with various volume Lipofectamine 2000 and different concentration FITC-labeled Oligo to optimize the transfection condition of Dnmt1s-siRNA, and the combination of 1.5 μl or 2 μl Lipo 2000 and 50 nM or 100 nM FITC-Oligo resulted in the high integrated optical density of FITC (Supplementary Figure 5A-5C), however, compared with the control group, 2 μl Lipo 2000 significantly reduced the cell number of PFFs (Supplementary Figure 5D, 1.58×10^4^ vs 2.02×10^4^, P<0.05), thus, a combination of 1.5 μl Lipo 2000 and 50 nM siRNA could be optimal to knock Dnmt1s down in donor cells. When Dnmt1s-siRNA was transfected into PFFs, Dnmt1s transcription was significantly downregulated with the increasing time of culture (Figure 3A and Supplementary Figure 6, 55.65%, 40.02%, 20.33%, 15.35% or 16.59% at 9 h, 18 h, 36 h, 54 h or 72 h vs 100.10% at 0 h, respectively, P<0.05) and the high reduction occurred at 36 h, 54 h or 72 h posttransfection, however, Dnmt1s knockdown for 54 h or 72 h significantly reduced the cell number of PFFs (Figure 3B, 4.03×10^4^ in the siRNA-positive group vs 5.48×10^4^ or 5.13×10^4^ in the control or siRNA-negative group for 54 h, or 4.63×10^4^ vs 6.90×10^4^ or 6.30×10^4^ for 72 h, respectively, P<0.05) and the fusion rate of reconstructed embryos (Figure 3C, 55.25% in the siRNA-positive group vs 69.08% or 66.43% in the control or siRNA-negative group for 54 h, or 31.54% vs 76.54% or 69.29% for 72 h, respectively, P<0.05). Together, donor cells with Dnmt1s knockdown for 36 h were suitable for SCNT.

When these Dnmt1s-knockdown donor cells were used, the blastocyst rate in the siRNA-positive group was significantly higher than that in the control or siRNA-negative group (Figure 3D and Table 1, 31.33% vs 20.37% or 21.50%, P<0.05). Notably, the rate of SCNT embryos arrested before ZGA in the siRNA-positive group were significantly reduced (Figure 3E, 30.41% vs 45.37% or 46.40% in the control or siRNA-negative group, P<0.05). These results suggested that Dnmt1s in donor cells can be effectively removed by siRNA, and Dnmt1s removal from donor cells improved the development of SCNT embryos.

**Table 1:** Development of SCNT embryos derived from donor cells with Dnmt1s knockdown

Group	No. embryoscultured (Rep.)	No. embryos cleaved(% ± SEM)	No. blastocysts(% ± SEM)	Blastocystcell numbers(mean ± SEM) *
control	163 (5)	138 (84.75 ± 1.98)	33 (20.37 ± 1.15)^a^	35 ± 2 (n=32)
siRNA- negative	167 (5)	139 (83.23 ± 1.43)	36 (21.50 ± 1.06)^a^	34 ± 3 (n=34)
siRNA- positive	176 (5)	153 (87.28 ± 1.73)	55 (31.33 ± 1.49)^b^	38 ± 3 (n=29)

*Blastocyst cell numbers, less than 16, were not included, and 25 blastocysts in the siRNA-positive group were used for molecular analysis.

^a-b^Values in the same column with different superscripts differ significantly (P < 0.05).

### Dnmt1s knockdown in donor cells was benefit for SCNT-mediated DNA methylation reprogramming during ZGA and further in blastocysts

**Figure 4:**
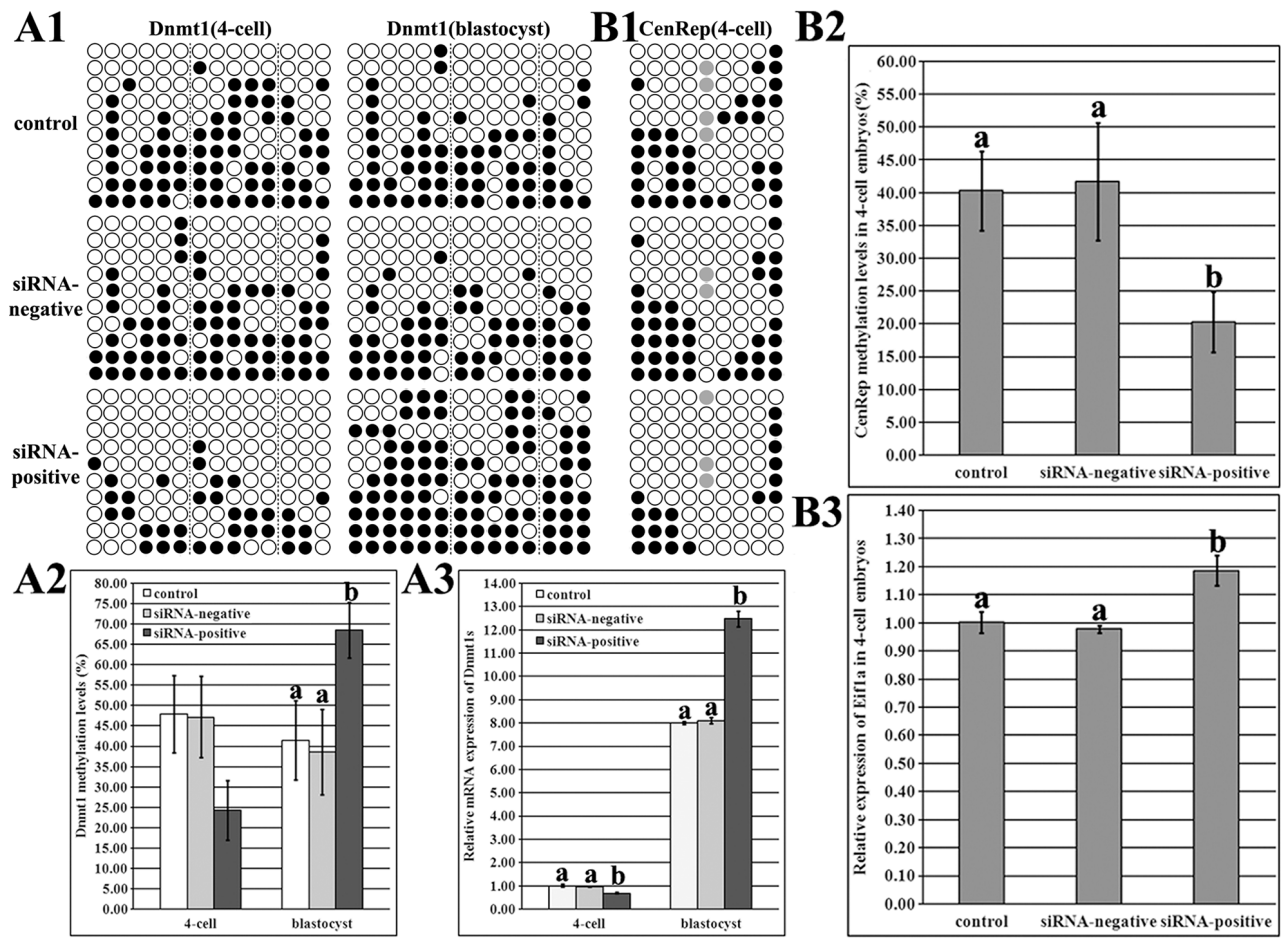
Dnmt1s and genome DNA methylation and expression levels in SCNT embryos after Dnmt1s knockdown in donor cells **(A1)**, DNA methylation statuses of Dnmt1s at the 4-cell and blastocyst stages in the control, siRNA-negative or siRNA-positive group, **(A2)**, DNA methylation levels of Dnmt1s at the 4-cell and blastocyst stages in the control, siRNA-negative or siRNA-positive group, **(A3)**, relative Dnmt1s expression levels at the 4-cell and blastocyst stages in the control, siRNA-negative or siRNA-positive group, **(B1)**, genomic DNA methylation status at the 4-cell stage in the control, siRNA-negative or siRNA-positive group, **(B2)**, genomic DNA methylation level at the 4-cell stage in the control, siRNA-negative or siRNA-positive group, and, **(B3)**, relative Eif1a expression level at the 4-cell stage in the control, siRNA-negative or siRNA-positive group. Dnmt1s knockdown in donor cells improved Dnmt1s and genome DNA methylation reprogramming and expression levels in SCNT embryos. Black or white circles indicate methylated or unmethylated CpG sites, respectively, and gray circles represent mutated and/or single nucleotide polymorphism (SNP) variation at certain CpG sites. The expression abundance of Dnmt1s (A3) or Eif1a (B3) at the 4-cell stage in the control group was considered to be the control. The data were expressed as mean ± SEM. ^a-b^Values at a given stage with different superscripts differ significantly (P<0.05).

As shown in Figure 4, compared with those in the control or siRNA-negative group, DNA methylation level of Dnmt1s was lower, and Dnmt1s transcription was significantly downregulated during ZGA (Figure 4A1-4A3, P<0.05), responding to the reduced arrest of SCNT embryos, and significantly higher levels of Dnmt1s methylation and expression were observed at the blastocyst stage in the siRNA-positive group (Figure 4A1-4A3, P<0.05), indicating that zygotic Dnmt1s could be essential for the subsequent development of SCNT embryos after ZGA. Thus, Dnmt1s knockdown in donor cells improved itself reconstruction of DNA methylation and transcription during ZGA and further in blastocysts.

As for genomic methylation reprogramming, the siRNA-positive group took on the significantly lower methylation level and higher Eif1a expression during ZGA in comparison with the control or siRNA-negative group (Figure 4B1-4B3, P<0.05), suggesting that Dnmt1s in donor cells blocks the development of SCNT embryos through disrupting DNA methylation reprogramming.

**Figure 5:**
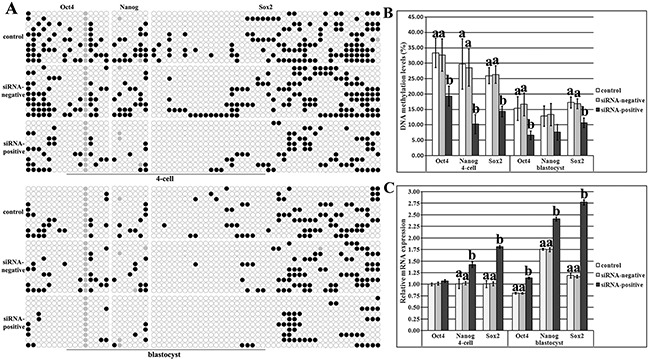
DNA methylation and expression levels of pluripotent genes in SCNT embryos after Dnmt1s knockdown in donor cells **(A)**, DNA methylation statuses of Oct4, Nanog and Sox2 at the 4-cell and blastocyst stages in the control, siRNA-negative or siRNA-positive group, **(B)**, DNA methylation levels of Oct4, Nanog and Sox2 at the 4-cell and blastocyst stages in the control, siRNA-negative or siRNA-positive group, and, **(C)**, relative expression levels of Oct4, Nanog and Sox2 at the 4-cell and blastocyst stages in the control, siRNA-negative or siRNA-positive group. Dnmt1s knockdown in donor cells promoted DNA methylation reprogramming and expression of Oct4, Nanog and Sox2 in SCNT embryos. Black or white circles indicate methylated or unmethylated CpG sites, respectively, and gray circles represent mutated and/or single nucleotide polymorphism (SNP) variation at certain CpG sites. The expression abundance of Oct4, Nanog or Sox2 at the 4-cell stage in the control group was considered to be the control. The data were expressed as mean ± SEM. ^a-b^Values for a given gene at a given stage with different superscripts differ significantly (P<0.05).

Then, DNA methylation and expression patterns of pluripotent genes (Oct4, Nanog and Sox2) were also examined. The distribution of CpG sites and methylation statuses of different CpG regions in the upstream of Sox2 TSS were first analyzed, and according to the methylation statuses and expression patterns in sperms, oocytes, PFFs and IVF blastocysts, Region II including 44 CpG sites could represent the DMR of Sox2 (Supplementary Figure 7). After Dnmt1s knockdown in donor cells, SCNT embryos displayed the significantly downregulated DNA methylation levels of Oct4, Nanog and Sox2 and upregulated expression of Nanog and Sox2 during ZGA in the siRNA-positive group compared with the control or siRNA-negative group, further leading to the significantly reduced Oct4 and Sox2 methylation levels and increased Oct4, Nanog and Sox2 expression in blastocysts (Figure 5, P<0.05), and more, the transcription levels of Dnmt3a, Cdx2, ATP1b1 and Bcl2l1/Bax were also significantly improved in blastocysts (Supplementary Figure 8, P<0.05). Therefore, donor cell Dnmt1s removal remarkably promoted the activation of genes required for embryonic development by improving DNA methylation reprogramming in SCNT embryos.

Overall, when Dnmt1s was effectively knocked down in donor cells, DNA methylation levels of Dnmt1s, genome and genes required for embryonic development were not obviously altered in PFFs (Supplementary Figure 9), but significantly improved in SCNT embryos, revealing that Dnmt1s in donor cells is a barrier to SCNT-mediated DNA methylation reprogramming.

## DISCUSSION

**Figure 6:**
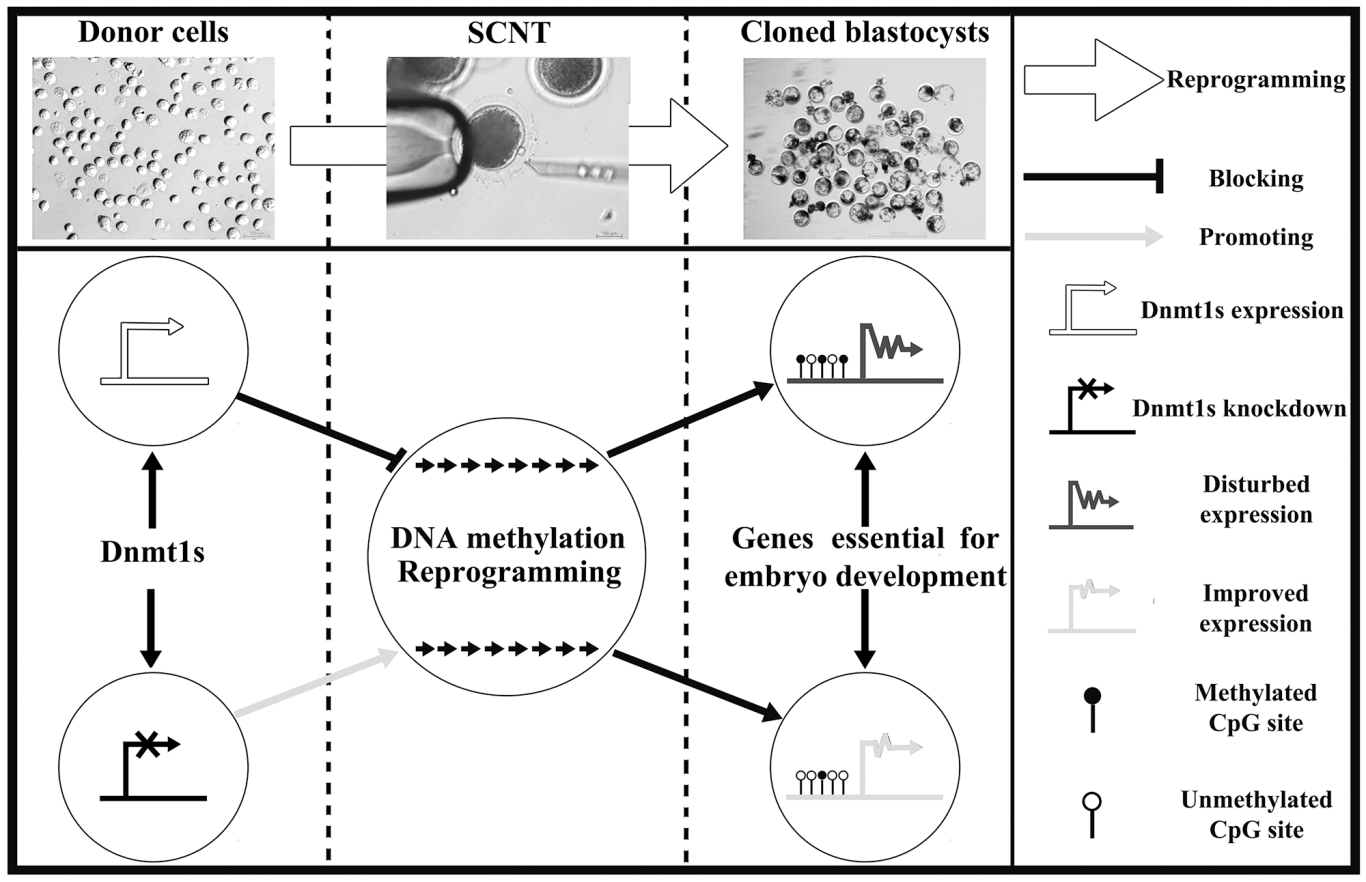
The potential mechanism of donor cell Dnmt1s blocking SCNT-mediated DNA methylation reprogramming Dnmt1s knockdown in donor cells improved the development of SCNT embryos by facilitating DNA methylation reprogramming and the expression of genes required for embryonic development.

Generally, incomplete DNA methylation reprogramming induced by SCNT can be due to the repressive factors present in donor cells [6]. Here, our results demonstrated that Dnmt1s in donor cells serves as a critical barrier for ZGA and genomic methylation reprogramming, leading to the developmental arrest of SCNT embryos, and removal of this barrier by siRNA enhances DNA methylation reprogramming, allows the efficient activation of genes required for embryonic development, and thus improves the SCNT efficiency (Figure 6).

It has been reported that Dnmt1 expression is disrupted in SCNT embryos [19, 20]. Here, our study showed that SCNT embryos displayed the higher Dnmt1 level during ZGA, and coincidentally, incomplete genomic methylation reprogramming and the developmental block were associated with this expression pattern. Usually, the failed genomic methylation reprogramming leads to the inefficient activation of genes essential for embryonic development [8], resulting in the low SCNT efficiency, while, the improved Dnmt1 expression can enhance genomic methylation reprogramming and the development of SCNT embryos [17]. So, these data indicate that the developmental arrest of SCNT embryos could be the consequence of the aberrant Dnmt1 expression.

Currently, no evidence exists that which isoform blocks the development of SCNT embryos. Here, we identified the DMR of Dnmt1o or Dnmt1s, basically consistent with the status in mice [14], and distinguished the individual expression pattern of Dnmt1o or Dnmt1s by the first exon. Interestingly, Dnmt1s, not Dnmt1o, displayed the aberrant DNA methylation and transcription patterns during the development of SCNT embryos. Indeed, our previous study has reported that Dnmt1 knockdown in SCNT embryos enhances the SCNT efficiency, further suggesting that the high Dnmt1s expression before ZGA can be the cause of the developmental arrest of SCNT embryos [15]. Moreover, Dnmt1 knockdown in donor cells contributes to the developmental efficiency of SCNT embryos [12, 13]. In combination with the incomplete genomic methylation reprogramming during ZGA, Dnmt1s deriving from donor cells is speculated to perpetuate the high methylation status of donor cells and impede the SCNT efficiency.

To enhance the development of SCNT embryos, Dnmt1s in donor cells should be removed. Here, siRNA was applied, as Dnmt1 inhibitors may be off target and Dnmt1 knockout leads to embryonic lethality, and RNAi technology has been employed to reduce Dnmt1 expression in somatic cells [21–23]. As Dnmt1 is essential for cell survival and maintenance of DNA methylation, and the knockdown efficiency and embryo fusion rate must be considered, notably, only Dnmt1s knockdown with 36 h was adopted, though various time points were detected and Dnmt1s was effectively reduced in donor cells. After Dnmt1s removal, the developmental arrest of SCNT embryos was obviously overcome. Moreover, Dnmt1s deletion facilitated the subsequent SCNT embryo development. Thus, Dnmt1s in donor cells can be markedly removed by siRNA, benefiting the SCNT efficiency.

It is known that Dnmt1s functions as the maintenance of DNA methylation, then, the elevated genomic methylation level during ZGA should be the cause of the presence of Dnmt1s, brought by donor cells into SCNT embryos. In support of this assumption, the mechanism underlying that Dnmt1s knockdown in donor cells enhanced the SCNT efficiency should be that DNA methylation reprogramming process is improved. In this study, it was observed that Dnmt1s removal from donor cells promoted itself reconstruction of DNA methylation and genomic methylation reprogramming during ZGA, suggesting that Dnmt1s existing in donor cells impeded the development of SCNT embryos through interfering with DNA demethylation progress. Additionally, DNA methylation patterns of pluripotent genes were also improved during ZGA after Dnmt1s removal, further supporting that Dnmt1s in donor cells disrupted DNA methylation reprogramming in SCNT embryos. During SCNT-mediated reprogramming, pluripotent genes are critical for the establishment and maintenance of pluripotency [24], thus, the efficient activation of pluripotent genes resulting from the improved DNA methylation patterns helped SCNT embryos to overcome the developmental arrest. And more, Dnmt1s removal from donor cells improved DNA methylation and expression patterns of embryo development related genes, including zygotic Dnmt1s, at the blastocyst stage of SCNT embryos, indicating that zygotic Dnmt1s could be essential for the subsequent development of SCNT embryos [23]. Taken together, these data clearly demonstrated that Dnmt1s knockdown in donor cells overcame the developmental arrest and enhanced the subsequent development of SCNT embryos by facilitating DNA methylation reprogramming and gene expression.

Of course, Dnmt1s may also interact with other enzymes or pathways to regulate the development of SCNT embryos [25–27], and other factors could also participate in the SCNT-mediated DNA methylation reprogramming [8, 28, 29], thus, the mechanism underlying that Dnmt1s knockdown enhanced the SCNT efficiency still needs to be clarified.

In conclusion, our results revealed that Dnmt1s in donor cells blocks the development of SCNT embryos, and its removal by siRNA markedly enhanced SCNT-mediated genomic and gene-specific DNA methylation reprogramming and the expression of genes required for embryonic development, thus, Dnmt1s in donor cells is considered as a critical barrier to SCNT-mediated DNA methylation reprogramming, impairing the development of SCNT embryos.

## MATERIALS AND METHODS

Chemicals were purchased from Sigma-Aldrich Corporation (St. Louis, MO, USA), and disposable and sterile plasticware was obtained from Nunclon (Roskilde, Denmark), unless otherwise stated. All experiments were approved by the Animal Care Commission of Qingdao Agricultural University according to animal welfare laws, guidelines and policies. All surgery was performed under sodium pentobarbital anaesthesia, and all efforts were made to minimize suffering

### Donor cell culture

Donor cell culture has been described previously [30]. Briefly, porcine fetuses were obtained from a sow at day 35 of pregnancy after the sow was anaesthetized and sacrificed, then porcine fetal fibroblasts (PFFs) were isolated from the fetuses under sodium pentobarbital anaesthesia. After removal of fetal head, internal organs and limbs, the remaining tissues were finely minced into pieces, digested with 0.25% trypsin-0.04% ethylenediaminetetraacetic acid solution (GIBCO), and then dispersed in high glucose enriched Dulbecco's modified Eagle's medium (DMEM, GIBCO) containing 10% fetal bovine serum (FBS, GIBCO) and 1% penicillin-streptomycin (GIBCO). The dispersed cells were centrifuged, resuspended and cultured in DMEM. Until confluence, PFFs were digested, centrifuged, resuspended in FBS containing 10% dimethyl sulfoxide and stored in liquid nitrogen until use. Prior to SCNT, PFFs were thawed, cultured and subsequently used in 3-5 passages.

### siRNA design, synthesis and transfection

The design and synthesis of Dnmt1 specific siRNA have been reported in our study [16]. According to the requirement of Invitrogen Block-iT RNAi Designer and the information of Dnmt1s mRNA sequence, the stealth siRNA related to Dnmt1s cytosine-C5 specific DNA methylase domain was designed and synthesized (Invitrogen), and the sequence was GATAAGAAGTTTGTCAGCAACATCA. The negative sequence was GATGAAGGTTTGACTCAACAAATCA. Then, siRNAs were dissolved with Rnase free H_2_O to the concentration of 20 μM.

To determine the optimal transfection condition, various combinations of siRNA concentrations (25 nM, 50 nM and 100 nM FITC labeled nonsilencing siRNA, FITC-Oligo, Invitrogen) and transfection reagent volumes (0.5 μl, 1 μl, 1.5μl and 2 μl Lipofectamine 2000, Lipo 2000, Invitrogen) were examined. Before transfection, PFFs were cultured in 400 μl Opti-MEM (GIBCO), 0.5 μl, 1 μl, 1.5μl or 2 μl Lipo 2000 was added into Opti-MEM and incubated at room temperature for 5 min, 20 μM FITC-Oligo was diluted into 250 nM, 500 nM or 1000 nM with Opti-MEM, then, 100 μl FITC-Oligo-Lipo 2000 complexes was obtained through a mixture of 50 μl Opti-MEM with 250 nM, 500 nM or 1000 nM FITC-Oligo and 50 μl Opti-MEM with 0.5 μl, 1 μl, 1.5μl or 2 μl Lipo 2000, incubated at room temperature for 30 min and added into each 24-well culture plate with PFFs and 400 μl Opti-MEM. After 6 h, the medium including FITC-Oligo-Lipo 2000 complexes was replaced by DMEM containing 10% FBS and 1% penicillin-streptomycin. And, 36 h posttransfection, PFFs were observed and photographed under a fluorescence microscope, and the fluorescence intensities were analyzed using Image Pro Plus. Then, Dnmt1s specific siRNA was transfected into PFFs under the optimal condition. The interference efficiency was examined at 9 h, 18 h, 36 h, 54 h or 72 h posttransfection, respectively. The negative siRNA with the same amount was transfected as a control.

To measure cell proliferation after siRNA transfection, PFFs were harvested at 9 h, 18 h, 36 h, 54 h or 72 h, respectively, and the cell number at every time point was determined with a hemacytometer. Then, the siRNA transfection manner with Dnmt1s significant knockdown but no effect of cell proliferation was applied in the subsequent experiments.

### Oocyte *in vitro* maturation

Oocyte maturation has been reported [30]. Briefly, porcine ovaries were collected from a local slaughterhouse. Just after exposure, ovaries were placed into physiological saline with antibiotics at 37 °C and transported to the laboratory. Follicles were aspirated, and follicular contents were washed with HEPES-buffered Tyrode's lactate. Cumulus-oocyte complexes (COCs) were recovered and cultured in maturation medium. After 42 h, COCs were vortexed in hyaluronidase for 30 sec to remove cumulus cells. Only oocytes with the visible polar body, regular morphology and homogenous cytoplasm were used.

### IVF and SCNT, and embryo culture

The procedures for IVF and SCNT have been described in our reports [15, 30]. Briefly, for IVF, the semen was incubated and washed in DPBS supplemented with BSA. The spermatozoa were diluted with modified Tris-buffered medium (mTBM) to the appropriate concentration. Matured oocytes were washed in mTBM, transferred into fertilization medium and co-incubated with spermatozoa. Then, the embryos were washed and cultured in porcine zygote medium-3 (PZM-3) for the subsequent development. For SCNT, matured oocytes and donor cells were placed into manipulation medium. After oocyte enucleation, donor cells were placed into the perivitelline space. Fusion and activation of the cell-cytoplast complexes were induced by electroporation. Then, the reconstructed embryos were cultured in PZM-3 for the subsequent development.

### Embryo development and collection

The fusion, cleavage and blastocyst rates were evaluated at 0.5 h, 48 h and 156 h postactivation, respectively.

For blastocyst cell number, embryos at 156 h postactivation were treated with acidic Tyrode's solution to remove zona pellucida, fixed in 4% paraformaldehyde for 30 min, and stained in DPBS containing 10 μg/ml Hoechst 33342 for 5 min in the dark. After staining, cloned embryos were washed and mounted on slides. Then, blastocyst cell number was examined under ultraviolet light from a fluorescence microscope.

For embryo collection, 1-cell, 2-cell, 4-cell, 8-cell and blastocyst embryos in each group were collected at 6 h, 24 h, 48 h, 72 h and 156 h, respectively.

### Quantitative real-time PCR

Measurement of gene expression with quantitative real-time PCR has been applied in our studies [19, 30]. Briefly, total RNA was extracted from 10^4^ PFFs, 50 MII oocytes or 50 pooled embryos at each stage using an RNeasy Mini Kit (Qiagen) according to the manufacturer's instructions, and the elution volume was 50 μl. Reverse transcription was performed using a PrimeScript RT Reagent Kit (TaKaRa). The 100 μl reaction volume contained 20 μl 5×PrimeScript Buffer, 5 μl PrimeScript RT Enzyme Mix I, 5 μl Oligo dT Primer (50 μM), 5 μl Random 6 mers (100 μM), 50 μl Total RNA and 15 μl RNase Free dH_2_O. The reaction condition was 37 °C for 15 min and 85 °C for 5 sec, and the cDNA was stored at -20 °C until use. For quantitative real-time PCR, reactions were performed in 96-well optical reaction plates (Applied Biosystems) using SYBR Premix ExTaq II (TaKaRa) and a 7500 Real-Time PCR System (Applied Biosystems). Each reaction mixture (20 μl) contained 2 μl cDNA solution, 10 μl 2×SYBR Premix Ex Taq II, 1.6 μl PCR primer (10 μM), 0.4 μl ROX Reference Dye II (50×) and 6 μl dH_2_O. Thermal cycling conditions were 95 °C for 30 sec, 40 two-step cycles of 95 °C for 5 sec and 60 °C for 34 sec, and finally a dissociation stage consisting of 95 °C for 15 sec, 60 °C for 1 min and 95 °C for 15 sec. For each sample, the cycle threshold (CT) values were obtained from three replicates. The primers used for amplification of target and internal reference genes were presented in Supplementary Table 1. The relative expression levels of target genes were analyzed using the 2^−ΔΔCT^ method.

### Bisulfite sequencing

Bisulfite sequencing has been reported [15, 17]. Briefly, pooled samples were digested with Proteinase K (PK) and treated with sodium bisulfite to convert all unmethylated cytosine to uracil using an EZ DNA Methylation-Direct Kit (Zymo Research). For semen, the sperm was collected by centrifugation, washed in SMB solution (10 mM Tris-HCl, 10 mM EDTA, 50 mM NaCl and 2% SDS) and incubated in SMB solution supplemented with 40 mM dithiothreitol and 0.3 mg/ml PK at 56 °C for 1 h. For samples of 10^4^ PFFs, 800 MII oocytes and 200, 100, 200, 25 and 25 pooled zona pellucida-removed embryos at the 1-cell, 2-cell, 4-cell, 8-cell and blastocyst stages, respectively, in each group, digestion was performed in M-Digestion Buffer supplemented with PK at 50 °C for 20 min. After digestion, a CT (cytosine to thymine) conversion reagent was added at 98 °C for 10 min and 64 °C for 2.5 h. Then, the samples were desalted, purified and diluted with M-Elution Buffer. Subsequently, nested PCR was carried out to amplify the target regions of CenRep, Dnmt1, Oct4, Nanog and Sox2 using the primers described in Supplementary Table 2 and Hot Start Taq Polymerase (TaKaRa) with a profile of 94 °C for 5 min, 45 cycles of 94 °C for 30 sec, the optimal primer annealing temperature for 30 sec and 72 °C for 1 min, followed by 72 °C for 10 min. Products from the first amplification reaction were used in the second PCR reaction. Then, the amplified products were verified by electrophoresis and purified using an Agarose Gel DNA Purification Kit (TaKaRa). The purified fragments were cloned into pMD18-T Vectors (TaKaRa) and subjected to sequence analysis.

### Statistical analysis

Differences in data (mean ± SEM) were analyzed with the SPSS statistical software. Statistical analysis of data concerning embryo development, DNA methylation, gene expression and cell proliferation were performed with one-way ANOVA when there were three or more groups or t-test for two groups. For all analyses, differences were considered to be statistically significant when P<0.05.

## SUPPLEMENTARY MATERIALS FIGURES AND TABLES





## Abbreviations

SCNT: somatic cell nuclear transfer
IVF: *in vitro* fertilization
ZGA: zygotic gene activation
DMR: differentially methylated region
PFFs: porcine fetal fibroblasts
FBS: fetal bovine serum
DMEM: Dulbecco's modified Eagle's medium
PK: Proteinase K
SNP: single nucleotide polymorphism
COCs: cumulus-oocyte complexes
mTBM: modified Tris-buffered medium.

## References

[1] Yamanaka S, Blau HM (2010). Nuclear reprogramming to a pluripotent state by three approaches. Nature.

[2] Galli C, Lagutina I, Perota A, Colleoni S, Duchi R, Lucchini F, Lazzari G (2012). Somatic Cell Nuclear Transfer and Transgenesis in Large Animals: Current and Future Insights. Reproduction in Domestic Animals.

[3] Keefer CL Artificial cloning of domestic animals. Proceedings of the National Academy of Sciences.

[4] Loi P, Iuso D, Czernik M, Ogura A (2016). A New, Dynamic Era for Somatic Cell Nuclear Transfer?. Trends in Biotechnology.

[5] Niemann H (2016). Epigenetic reprogramming in mammalian species after SCNT-based cloning. Theriogenology.

[6] Sepulveda-Rincon LP, Solanas Edel L, Serrano-Revuelta E, Ruddick L, Maalouf WE, Beaujean N (2016). Early epigenetic reprogramming in fertilized, cloned, and parthenogenetic embryos. Theriogenology.

[7] Niemann H, Carnwath JW, Herrmann D, Wieczorek G, Lemme E, Lucas-Hahn A, Olek S (2010). DNA Methylation Patterns Reflect Epigenetic Reprogramming in Bovine Embryos. Cellular Reprogramming.

[8] Peat JR, Reik W (2012). Incomplete methylation reprogramming in SCNT embryos. Nature Genetics.

[9] Zhang S, Chen X, Wang F, An X, Tang B, Zhang X, Sun L, Li Z (2016). Aberrant DNA methylation reprogramming in bovine SCNT preimplantation embryos. Sci Rep.

[10] Matoba S, Liu Y, Lu F, Iwabuchi Kumiko A, Shen L, Inoue A, Zhang Y (2014). Embryonic Development following Somatic Cell Nuclear Transfer Impeded by Persisting Histone Methylation. Cell.

[11] Liao HF, Mo CF, Wu SC, Cheng DH, Yu CY, Chang KW, Kao TH, Lu CW, Pinskaya M, Morillon A, Lin SS, Cheng WTK, Bourc’his D (2015). Dnmt3l-knockout donor cells improve somatic cell nuclear transfer reprogramming efficiency. Reproduction.

[12] Selokar NL, Saini M, Agrawal H, Palta P, Chauhan MS, Manik R, Singla SK (2015). Downregulation of DNA Methyltransferase 1 in Zona-Free Cloned Buffalo (Bubalus bubalis) Embryos by Small Interefering RNA Improves *In Vitro* Development But Does Not Alter DNA Methylation Level. Cellular Reprogramming.

[13] Yamanaka K, Sakatani M, Kubota K, Balboula AZ, Sawai K, Takahashi M (2011). Effects of downregulating DNA methyltransferase 1 transcript by RNA interference on DNA methylation status of the satellite I region and *in vitro* development of bovine somatic cell nuclear transfer embryos. J Reprod Dev.

[14] Ko YG, Nishino K, Hattori N, Arai Y, Tanaka S, Shiota K (2005). Stage-by-Stage Change in DNA Methylation Status of Dnmt1 Locus during Mouse Early Development. Journal of Biological Chemistry.

[15] Huan Y, Wu Z, Zhang J, Zhu J, Liu Z, Song X (2015). Epigenetic Modification Agents Improve Gene-Specific Methylation Reprogramming in Porcine Cloned Embryos. Plos One.

[16] Huan Y, Xie B, Liu S, Kong Q, Liu Z (2015). A Novel Role for DNA Methyltransferase 1 in Regulating Oocyte Cytoplasmic Maturation in Pigs. Plos One.

[17] Huan YJ, Zhu J, Wang HM, Wu ZF, Zhang JG, Xie BT, Li JY, Kong QR, Liu ZH, He HB (2014). Epigenetic modification agents improve genomic methylation reprogramming in porcine cloned embryos. J Reprod Dev.

[18] Huan Y, Wang H, Wu Z, Zhang J, Zhu J, Liu Z, He H (2015). Epigenetic Modification of Cloned Embryos Improves Nanog Reprogramming in Pigs. Cellular Reprogramming.

[19] Huan Y, Wang H, Wu Z, Zhang J, Liu Z, He H (2015). The expression patterns of DNA methylation reprogramming related genes are associated with the developmental competence of cloned embryos after zygotic genome activation in pigs. Gene Expression Patterns.

[20] Chung YG (2003). Abnormal Regulation of DNA Methyltransferase Expression in Cloned Mouse Embryos. Biology of Reproduction.

[21] Li E, Bestor TH, Jaenisch R (1992). Targeted mutation of the DNA methyltransferase gene results in embryonic lethality. Cell.

[22] Giraldo AM, Vaught TD, Fu L, Duncan AJ, Vance AM, Mendicino M, Ayares DL (2009). Gene expression pattern and downregulation of DNA methyltransferase 1 using siRNA in porcine somatic cells. Gene Expr.

[23] Golding MC, Williamson GL, Stroud TK, Westhusin ME, Long CR (2011). Examination of DNA methyltransferase expression in cloned embryos reveals an essential role for Dnmt1 in bovine development. Molecular Reproduction and Development.

[24] Bortvin A, Eggan K, Skaletsky H, Akutsu H, Berry DL, Yanagimachi R, Page DC, Jaenisch R (2003). Incomplete reactivation of Oct4-related genes in mouse embryos cloned from somatic nuclei. Development.

[25] Denis H, Ndlovu MN, Fuks F (2011). Regulation of mammalian DNA methyltransferases: a route to new mechanisms. EMBO reports.

[26] Di Ruscio A, Ebralidze AK, Benoukraf T, Amabile G, Goff LA, Terragni J, Figueroa ME, LL De Figueiredo Pontes, Alberich-Jorda M, Zhang P, Wu M, D’Alò F, Melnick A (2013). DNMT1-interacting RNAs block gene-specific DNA methylation. Nature.

[27] Mohan KN, Chaillet JR (2013). Cell and Molecular Biology of DNA Methyltransferase 1. Int Rev Cell Mol Biol.

[28] Ao X, Sa R, Wang J, Dao R, Wang H, Yu H (2016). Activation-induced cytidine deaminase selectively catalyzed active DNA demethylation in pluripotency gene and improved cell reprogramming in bovine SCNT embryo. Cytotechnology.

[29] Kwon D, Ji M, Lee S, Seo KW, Kang KS (2016). Reprogramming Enhancers in Somatic Cell Nuclear Transfer, iPSC Technology, and Direct Conversion. Stem Cell Rev.

[30] Huan YJ, Zhu J, Xie BT, Wang JY, Liu SC, Zhou Y, Kong QR, He HB, Liu ZH (2013). Treating Cloned Embryos, But Not Donor Cells, with 5-aza-2’-deoxycytidine Enhances the Developmental Competence of Porcine Cloned Embryos. J Reprod Dev.

