# Supplementary material for: Dnmt1s in donor cells is a barrier to SCNT-mediated DNA methylation reprogramming in pigs
Source: Oncotarget. 2017 Mar 23;8(21):34980–91. doi: 10.18632/oncotarget.16507 (PMC5471028; doi:10.18632/oncotarget.16507)
Supplement: Supplementary file 1 [file oncotarget-08-34980-s001.pdf]

Dnmt1s in donor cells is a barrier to SCNT-mediated DNA methylation reprogramming in pigs

SUPPLEMENTARY MATERIALS

SUPPLEMENTARY FIGURES AND TABLES

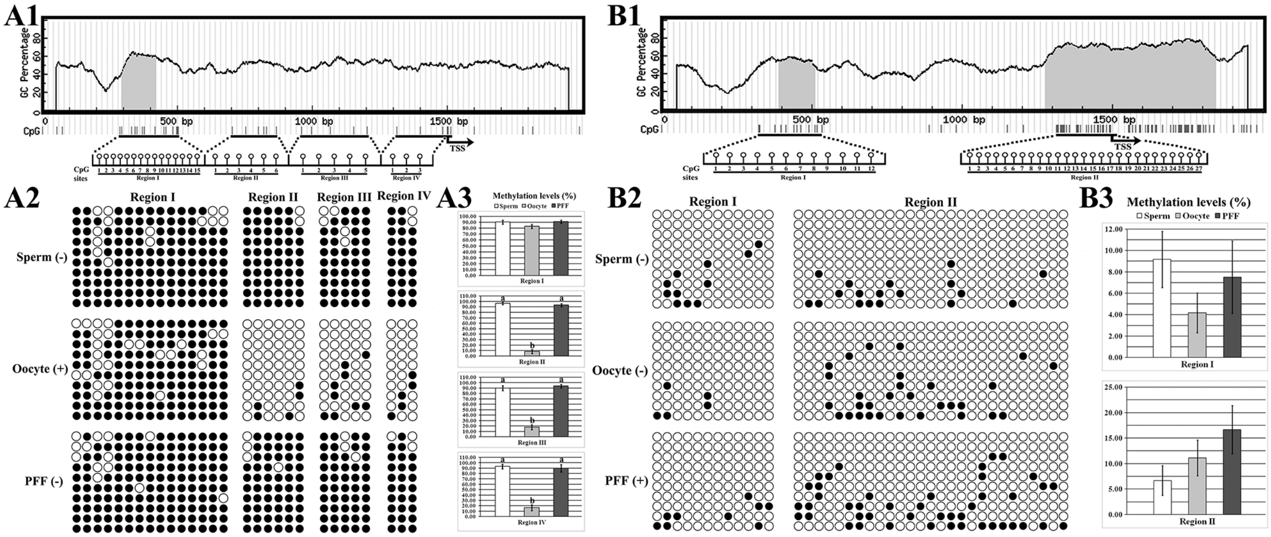

Supplementary Figure 1: Prediction and analysis of Dnmt1 methylation status. (A), prediction and analysis of Dnmt1o methylation statuses in sperms, oocytes and PFFs. **(A1)**, 29 CpG sites (15 in Region I, 6 in Region II, 5 in Region III and 3 in Region IV, respectively) were analyzed in the upstream sequence of Dnmt1o TSS by the MethPrimer program. **(B)**, prediction and analysis of Dnmt1s methylation statuses in sperms, oocytes and PFFs. B1, 39 CpG sites (12 in Region I and 27 in Region II, respectively) were analyzed in the upstream sequence of Dnmt1s TSS (located 9794 bp downstream of Dnmt1o TSS). **(A2)** and **(B2)**, the methylation statuses of different regions of Dnmt1o and Dnmt1s in sperms, oocytes and PFFs. **(A3)** and **(B3)**, the methylation levels of different regions of Dnmt1o and Dnmt1s in sperms, oocytes and PFFs. According to the expression levels and DNA methylation patterns of Dnmt1o and Dnmt1s in sperms, oocytes and PFFs, Region II, Region III and Region IV of Dnmt1o (total 14 CpG sites) could represent the methylation status of Dnmt1o or Dnmt1s. Black or white circles indicate methylated or unmethylated CpG sites, respectively. (+) represents gene expression, while (-) stands for no expression. The data were expressed as mean ± SEM. [a-b]Values in the same group with different superscripts differ significantly ($P<0.05$).

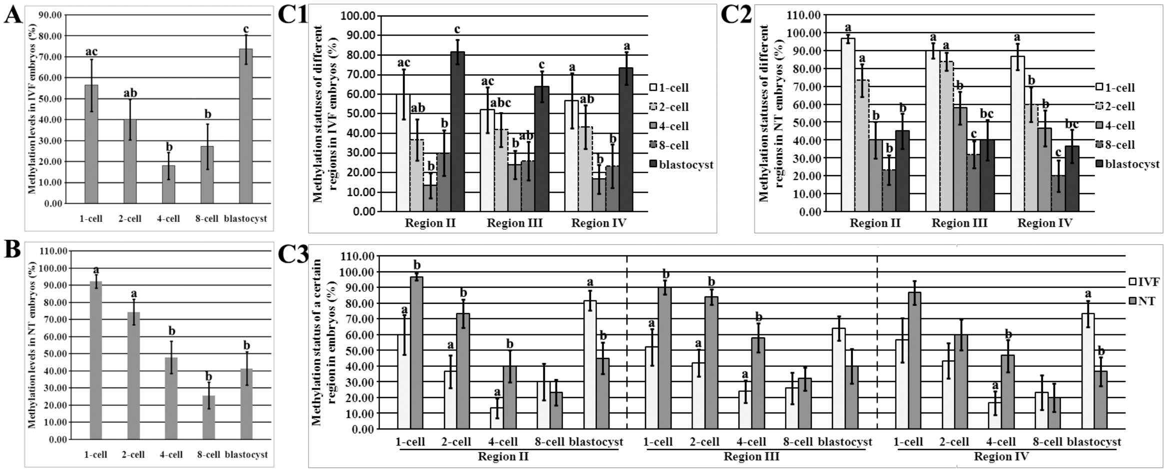

Supplementary Figure 2: Dnmt1 methylation levels in IVF and SCNT embryos. **(A)** and **(B)**, Dnmt1 methylation levels at the 1-cell, 2-cell, 4-cell, 8-cell and blastocyst stages of IVF and SCNT embryos. **(C1)** and **(C2)**, Dnmt1o Region II, Region III and Region IV methylation levels at the 1-cell, 2-cell, 4-cell, 8-cell and blastocyst stages of IVF and SCNT embryos. **(C3)**, a certain region methylation levels of Dnmt1o Region II, Region III and Region IV at the 1-cell, 2-cell, 4-cell, 8-cell and blastocyst stages between IVF and SCNT embryos. DNA methylation pattern in the individual Region II, Region III or Region IV was similar to the whole status of Dnmt1 in the IVF and SCNT embryos, and SCNT embryos displayed the disrupted Dnmt1 methylation pattern. The data were expressed as mean ± SEM. [a-c]Values in the same group or a certain region with different superscripts differ significantly (P<0.05).

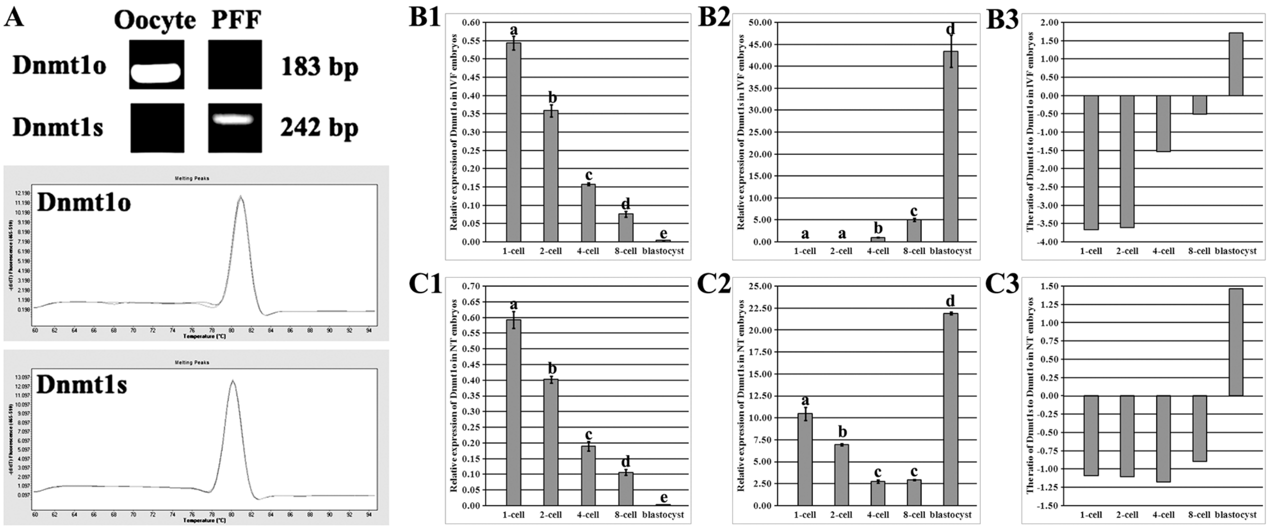

Supplementary Figure 3: Dnmt1o and Dnmt1s transcription levels in IVF and SCNT embryos. **(A)**, the amplified bands and melting curves of Dnmt1o and Dnmt1s. **(B1)** and **(B2)**, relative expression levels of Dnmt1o and Dnmt1s at the 1-cell, 2-cell, 4-cell, 8-cell and blastocyst stages of IVF embryos. **(B3)**, the ratio of Dnmt1s to Dnmt1o in IVF embryos. This ratio was the base 10 logarithm value. **(C1)** and **(C2)**, relative expression levels of Dnmt1o and Dnmt1s at the 1-cell, 2-cell, 4-cell, 8-cell and blastocyst stages of SCNT embryos. **(C3)**, the ratio of Dnmt1s to Dnmt1o in SCNT embryos. This ratio was the base 10 logarithm value. Different expression patterns of Dnmt1s were observed between IVF and SCNT embryos. The transcript abundance for Dnmt1o in MII oocytes or Dnmt1s in IVF 4-cell embryos was considered the control. The data were expressed as mean ± SEM. [a-e]Values for a given gene in the same group with different superscripts differ significantly ($p < 0.05$).

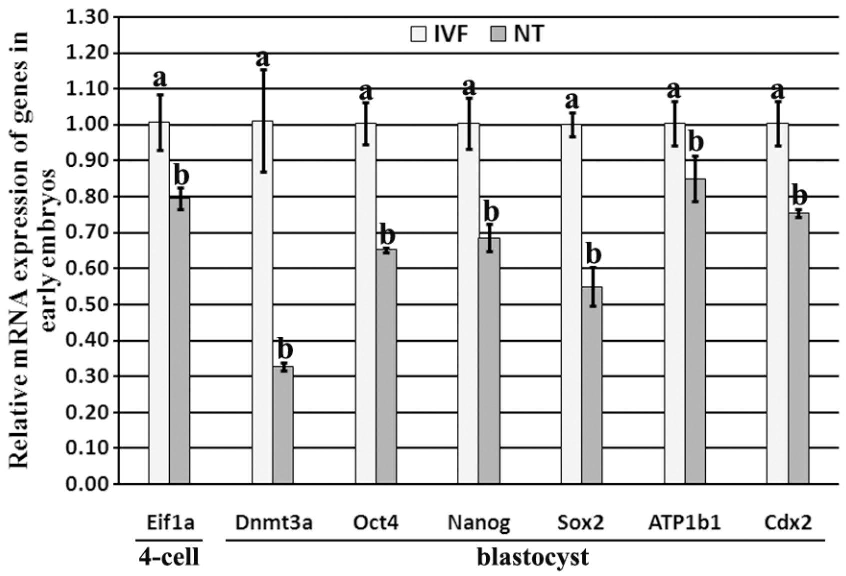

Supplementary Figure 4: Transcription levels of embryonic development related genes at the 4-cell and blastocyst stages of IVF and SCNT embryos. In comparison with IVF embryos, SCNT embryos displayed the obviously reduced expression of Eif1a at the 4-cell stage and Dnmt3a, Oct4, Nanog, Sox2, ATP1b1 and Cdx2 at the blastocyst stage. The transcript abundance for each gene in IVF embryos was considered the control. The data were expressed as mean ± SEM. [a-b]Values for a given gene with different superscripts differ significantly ($p < 0.05$).

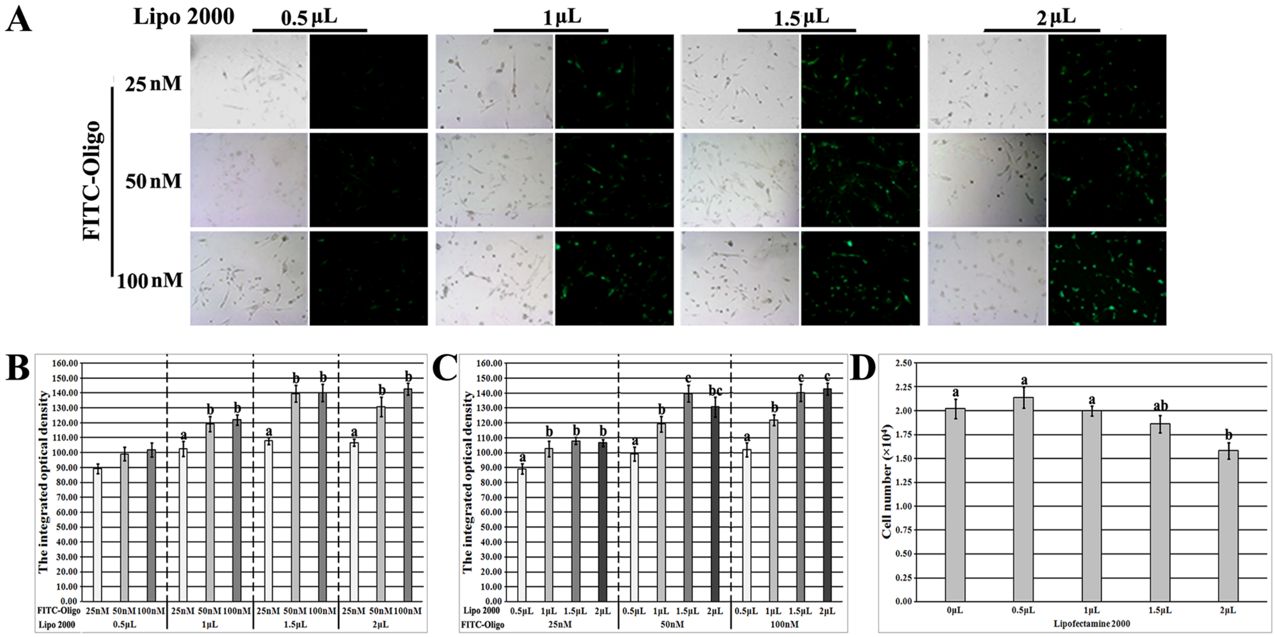

Supplementary Figure 5: The optimization of transfection condition using FITC-labeled Oligo. (A), light (left) and fluorescence (right) photographs of PFFs after 36 h posttransfection with various volume Lipofectamine 2000 (0.5 μl, 1 μl, 1.5 μl or 2 μl Lipo 2000) and different concentration FITC-labeled Oligo (25 nM, 50 nM or 100 nM FITC-Oligo). **(B)** and **(C)**, the integrated optical density of FITC after 36 h posttransfection at the various volume of Lipo 2000 and different concentration of FITC-Oligo. **(D)**, cell number of PFFs after 36 h posttransfection at different volumes of Lipo 2000. A combination of 1.5 μl Lipo 2000 and 50 nM siRNA resulted in the high integrated optical density of FITC. The data were expressed as mean ± SEM. [a-c]Values in a certain group with different superscripts differ significantly ($P<0.05$).

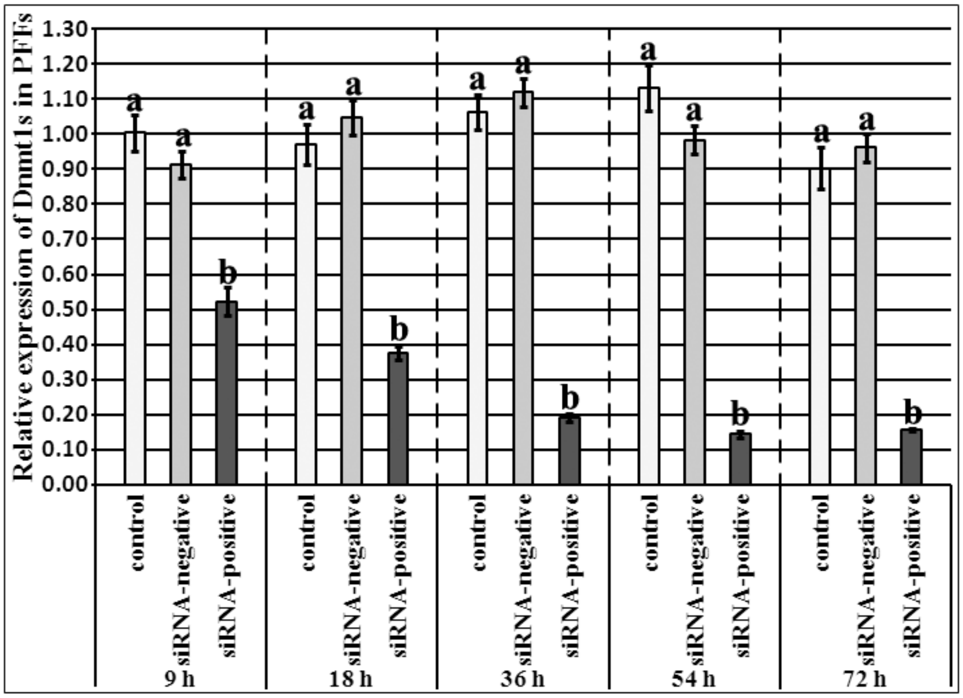

Supplementary Figure 6: Dnmt1s expression in PFFs after Dnmt1s-siRNA transfection. Dnmt1s-siRNA significantly reduced Dnmt1s transcription in PFFs at 9 h, 18 h, 36 h, 54 h or 72 h posttransfection. The data were expressed as mean ± SEM. [a-b]Values in a certain group with different superscripts differ significantly ($P<0.05$).

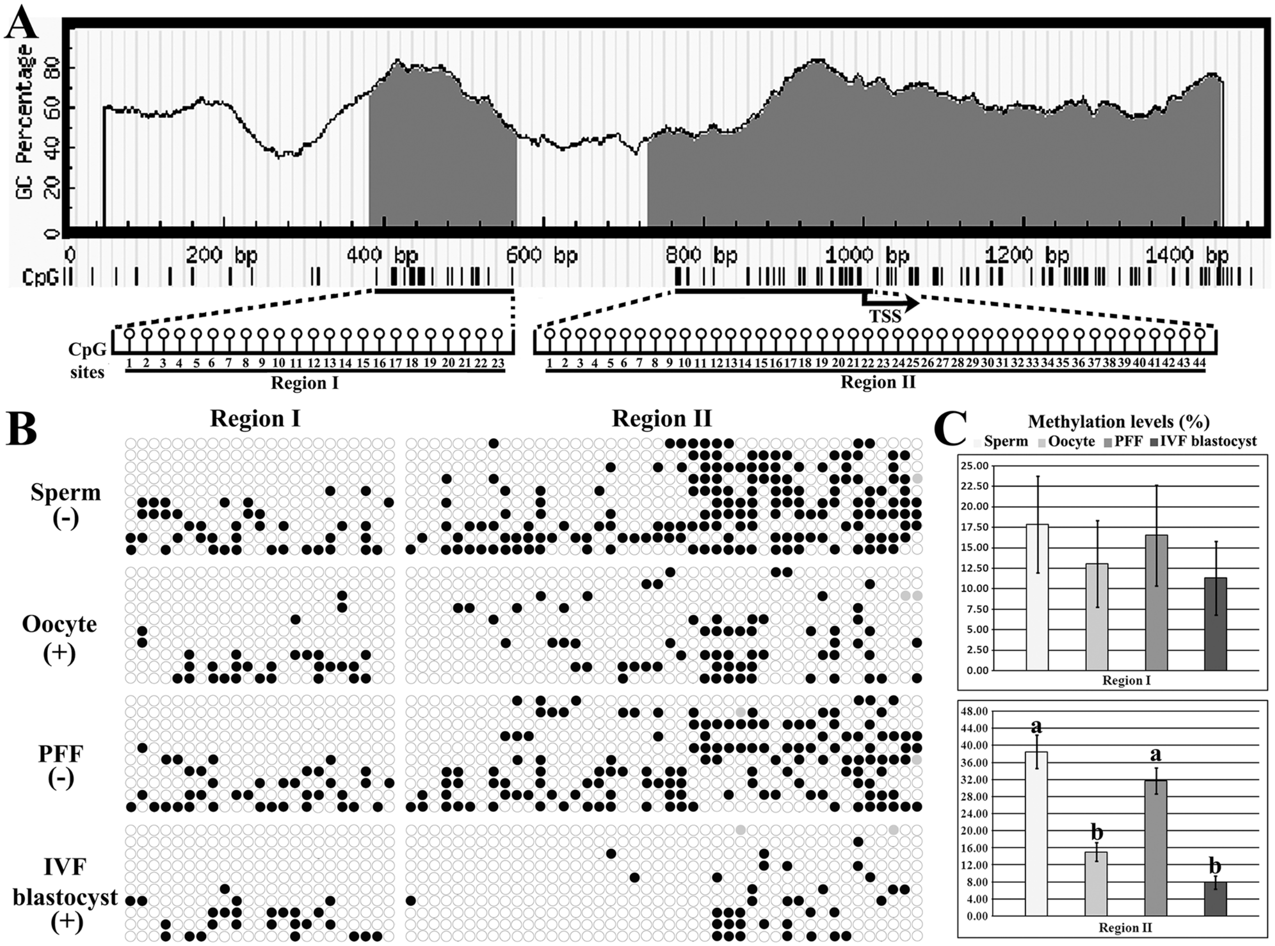

Supplementary Figure 7: Prediction and analysis of Sox2 methylation region. **(A)**, 67 CpG sites (23 in Region I and 44 in Region II, respectively) in the upstream of Sox2 TSS were analyzed by the MethPrimer program, **(B)**, methylation statuses of Sox2 Region I and Region II in sperms, oocytes, PFFs and IVF blastocysts, and, **(C)**, methylation levels of Sox2 Region I and Region II in sperms, oocytes, PFFs and IVF blastocysts. According to the expression levels and methylation patterns of Sox2 in sperms, oocytes, PFFs and IVF blastocysts, Region II could represent Sox2 methylation status. Black or white circles indicate methylated or unmethylated CpG sites, respectively, and gray circles represent mutated and/or single nucleotide polymorphism (SNP) variation at certain CpG sites. (+) represents gene expression, while (-) stands for no expression. The data were expressed as mean ± SEM. [a-b]Values for a given region with different superscripts differ significantly ($P<0.05$).

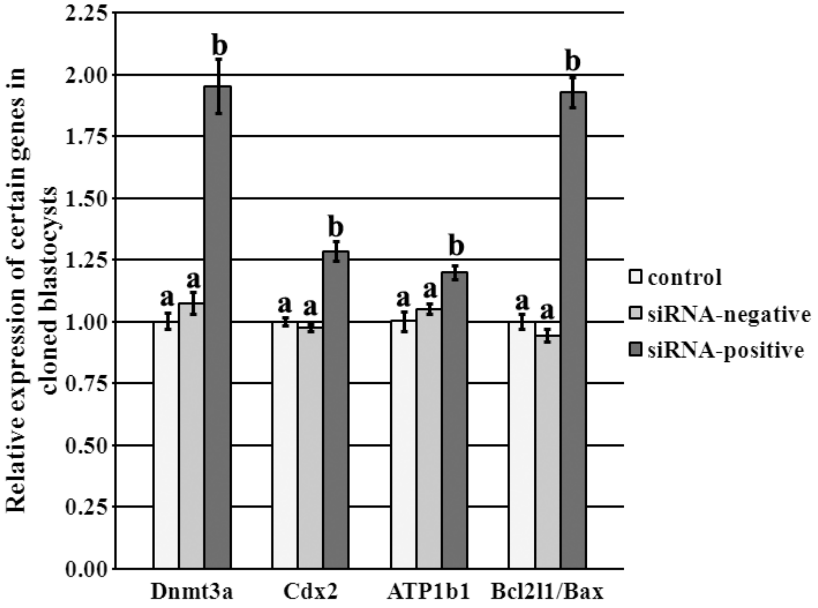

Supplementary Figure 8: Transcription levels of blastocyst quality related genes at the blastocyst stage in the control, siRNA-negative or siRNA-positive group. In comparison with the control or siRNA-negative group, the siRNA-positive group displayed the significantly higher expression of Dnmt3a, Cdx2, ATP1b1 and Bcl2l1/Bax. The transcript abundance for each gene in the control group was considered the control. The data were expressed as mean ± SEM. [a-b]Values for a given gene with different superscripts differ significantly ($p < 0.05$).

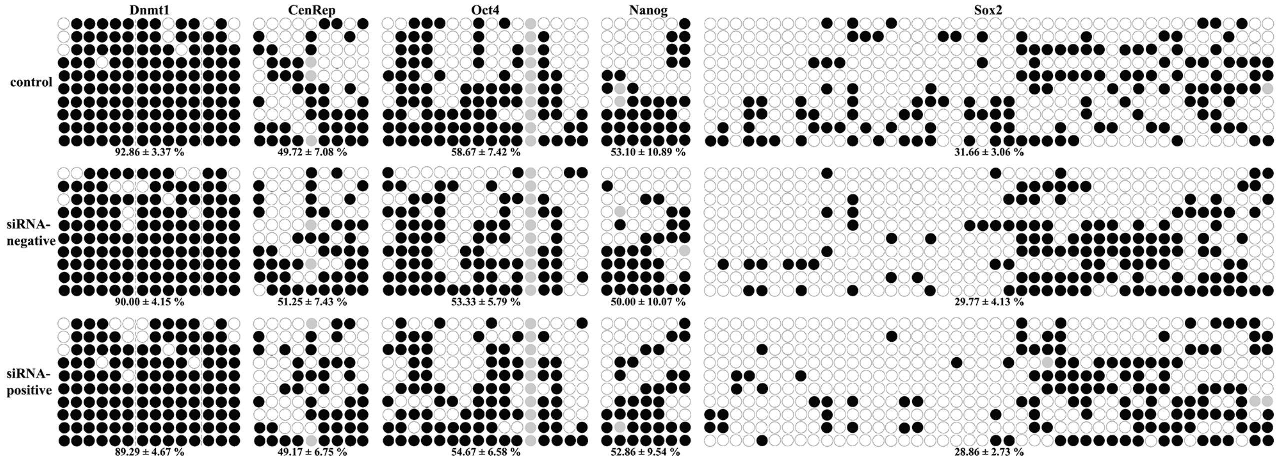

Supplementary Figure 9: DNA methylation statuses and levels of Dnmt1s, genome and pluripotent genes in PFFs after Dnmt1s knockdown. No significant differences of Dnmt1s, genome and pluripotent gene methylation were observed in PFFs after Dnmt1s knockdown. Black or white circles indicate methylated or unmethylated CpG sites, respectively, and gray circles represent mutated and/or single nucleotide polymorphism (SNP) variation at certain CpG sites. The data were expressed as mean ± SEM.

Supplementary Table 1: Detail of primers for quantitative real-time PCR

Gene	Primer sequence (5'-3')	Length (bp)	Accession number
Oct4	F: GAAGGTGTTCAGCCAAACGAC	185	NM_001113060
	R: CGATACTTGTCCGCTTTC		
Nanog	F: CCTCCATGGATCTGCTTATTC	209	NM_001129971
	R: CATCTGCTGGAGGCTGAGGT		
Sox2	F: AACCAGAAGAACAGCCCAGAC	155	NM_001123197
	R: TCCGACAAAAGTTTCCACTCG		
Dnmt1	F: GCGTCTTGCAGGCTGGTCAGTA	152	NM_001032355
	R: CTTCTTATCATCGACCACGACGCT		
Dnmt1s	F: CCAAGATGCCTGCCCGTAC	242	NM_001032355
	R: CAGGTAGCCCTCCTCTGATAATTC		
Dnmt1o	F: TGTGTTTTCACTTTGTGTCTTGGG	183	NM_001032355
	R: CAGGTAGCCCTCCTCTGATAATTC		
Eif1a	F: AGATGAGGCTAGAAGTCTGAAGGC	113	NM_001243218
	R: CAATGTCATCAAACTGGATTTCATC		
Dnmt3a	F: ATGTGGTTCGGAGACGGCAAGT	195	NM_001097437
	R: GCTCTCGTCGTTGTCATGGCA		
Cdx2	F: GACAAGGACGTGAGCATGTATCC	220	XM_003130908
	R: CGTAGCCGTTCCAGTCCTCG		
ATP1b1	F: CCACCAGGATTAACACAGATTCC	185	NM_001001542
	R: TCTTTGAGTTCGCTGGGCAC		
Bcl2l1	F: CTGGTGGTTGACTTTCTCTCCTAC	119	NM_214285
	R: GTTTCCGCTTCTGATTCAGTCC		
Bax	F: CAGTAACATGGAGCTGCAGAGG	159	AJ_606301
	R: GCCTTGAGCACCAGTTTACTGG		
β2m	F: CTTTCTACCTTCTGGTCCACACTG	116	NM_213978
	R: GTGGTCTCGATCCCACTTAACTATC		
18s	F: AATCTCGGGTGGCTGAACGC	143	NR_002170
	R: CCGTTCTTAGTTGGTGGAGCGAT		

Supplementary Table 2: Detail of primers for bisulfite sequencing.

See Supplementary File 1
