# Supplementary material for: Dnmt1s in donor cells is a barrier to SCNT-mediated DNA methylation reprogramming in pigs
Source: Oncotarget. 2017 Mar 23;8(21):34980–91. doi: 10.18632/oncotarget.16507 (PMC5471028; doi:10.18632/oncotarget.16507)
Supplement: Supplementary file 2 [file oncotarget-08-34980-s002.doc]

Table S2 Detail of primers for bisulfite sequencing

| Gene | Primer sequence (5'-3') | Primer annealing temperature  (°C) | Length (bp) | Accession number |
| --- | --- | --- | --- | --- |
| CenRep | F:GGTATTGTTGTTTGTTTGGTGATT | 55 | 231 | Z75640 |
|  | R:AAAATTTATTCCTCAAACCCAATTT |  |  |  |
| Dnmt1o (Region I) | Outer | 51 | 422 | NC_010444 |
|  | F:TAGTGGGAGTTTTTAATATAGGT |  |  |  |
|  | R:CCTAATAAAACTCAAATAACTCTC |  |  |  |
|  | Inner | 45 | 356 |  |
|  | F:AATAAAGTTGGAGGGAAAAA |  |  |  |
|  | R:TTTTTTTTACACACTCCCC |  |  |  |
| Dnmt1o (Region II) | Outer | 48 | 367 | NC_010444 |
|  | F:AAAGAAAATAGAGAGTTATTTGAG |  |  |  |
|  | R:TAAAAATTCCTACTATAATACAATA |  |  |  |
|  | Inner | 46 | 245 |  |
|  | F:TTTGATGGGATTTATTTATGAT |  |  |  |
|  | R:ATCTAACTAATAACCATAAAATTTC |  |  |  |
| Dnmt1o (Region III) | Outer | 52 | 397 | NC_010444 |
|  | F:GAAATTTTATGGTTATTAGTTAGA |  |  |  |
|  | R:CAAACACTATTCCTCCACC |  |  |  |
|  | Inner | 47 | 307 |  |
|  | F:AAAAATGTTGATTTGGGGT |  |  |  |
|  | R:CTAAACCCTTCAAATCCTTA |  |  |  |
| Dnmt1o (Region IV) | Outer | 50 | 342 | NC_010444 |
|  | F:AAGGGTTTAGGTGGAGGA |  |  |  |
|  | R:AAAAACAATAATCAAACCTCA |  |  |  |
|  | Inner | 47 | 266 |  |
|  | F:ATTGGGAATGTGGGGA |  |  |  |
|  | R:AAACATCACTTACCTCCCA |  |  |  |
| Dnmt1s (Region I) | Outer | 45 | 466 | NC_010444 |
|  | F:TTTTAAAAAAATATAAGTATAATTGA |  |  |  |
|  | R:TAACTTTACCACTACAATACCC |  |  |  |
|  | Inner | 47 | 389 |  |
|  | F:ATTTATGTTGTATAGGAAAGTGA |  |  |  |
|  | R:AAATTACACATACTACAACCTTAA |  |  |  |
| Dnmt1s (Region II) | Outer | 46 | 288 | NC_010444 |
|  | F:ATGAATGTTTTAGGTAAGGTT |  |  |  |
|  | R:CATCTTAAAAAATACAACAAAA |  |  |  |
|  | Inner | 47 | 235 |  |
|  | F:TTTAGTAAATAGAAGTTTGGATTAG |  |  |  |
|  | R:CACAAACAACCCTAACTTTTC |  |  |  |
| Oct4 | Outer  F:ATTAGATTTGTGTGAGGATTTGAGAG | 53 | 409 | NC_010449 |
|  | R:AAAACCCAATAAAACCAAAACTCTC |  |  |  |
|  | Inner  F:GAAGAGGGGTTTAATATTTGG | 50 | 288 |  |
|  | R:CCCAATCCCACCCACTAA |  |  |  |
| Nanog | Outer | 50 | 518 | EF_522119 |
|  | F:TGAATTGGAGATTTAAAGGAG |  |  |  |
|  | R:TAAAATCATAAAAATCTCCTCC |  |  |  |
|  | Inner | 52 | 498 |  |
|  | F:GGAGATTTAAAGGAGTTTTAGGTTAAGAAA |  |  |  |
|  | R:TCTCCTCCAAATATTAAAAATATCAAAAA |  |  |  |
| Sox2 (Region I) | Outer | 50 | 392 | NC_010455 |
|  | F:GTTGTGAGGGGGGATATAAAGG |  |  |  |
|  | R:CTAAAACTCAAACTTCTCTCCCTTTC |  |  |  |
|  | Inner | 58 | 381 |  |
|  | F:GGGGATATAAAGGTTTTTTAGTGGT |  |  |  |
|  | R:AAAACTCAAACTTCTCTCCCTTTCT |  |  |  |
| Sox2 (Region II) | Outer | 50 | 461 | NC_010455 |
|  | F:GTGTTTGTAAAAGGGGGAAAGTAG |  |  |  |
|  | R:CCGCAACAATAAAATTACCACC |  |  |  |
|  | Inner | 50 | 321 |  |
|  | F:AAGGGAGAGAAGTTTGAGTTTTAGG |  |  |  |
|  | R:AACTCCGTCTCCATCATATTATACATAC |  |  |  |
